# The use of tacrolimus for recurrent lupus enteritis: a case report

**DOI:** 10.1186/1752-1947-4-150

**Published:** 2010-05-24

**Authors:** Tsuyoshi Shirai, Yasuhiko Hirabayashi, Ryu Watanabe, Yumi Tajima, Hiroshi Fujii, Naruhiko Takasawa, Tomonori Ishii, Hideo Harigae

**Affiliations:** 1 Department of Rheumatology and Hematology, Tohoku University Graduate School of Medicine, Seiryo-cho, Aoba-ku, Sendai, 980-8574, Japan

## Abstract

**Introduction:**

Patients with lupus enteritis sometimes experience recurrence. In such cases, the addition of cyclophosphamide to the treatment regimen is recommended. However, an appropriate treatment has not been established in cases where cyclophosphamide failed to prevent the disease.

**Case presentation:**

An 18-year-old Japanese woman was admitted for a recurrence of lupus enteritis. One year before admission she was treated for lupus enteritis with high-dose corticosteroid together with intravenous cyclophosphamide pulse therapy. Upon admission, she was administered again with high-dose corticosteroid and her abdominal pain rapidly subsided. Tacrolimus was later used as an immunosuppressive agent and a complete remission has been maintained.

**Conclusion:**

Tacrolimus can be a useful agent for recurrent lupus enteritis that is resistant to conventional therapy.

## Introduction

Patients with systemic lupus erythematosus (SLE) occasionally present with acute abdominal symptoms. In some cases, abdominal symptoms can be the initial clinical presenting feature of SLE [1]. Lupus enteritis due to intestinal vasculitis is the most serious gastrointestinal complication of SLE presenting with acute abdominal symptoms [2]. During follow-up, patients with lupus enteritis sometimes experience recurrence after experiencing complete remission with the aid of high-dose corticosteroids [3]. In such cases, the addition of cyclophosphamide to the treatment regimen is recommended [4]. However, an appropriate treatment has not been established in cases where cyclophosphamide failed to prevent the occurrence of the disease. Here we present the case of a woman patient with refractory lupus enteritis who was treated successfully with tacrolimus but not with cyclophosphamide.

## Case presentation

An 18-year-old Japanese woman complaining of nausea, vomiting, abdominal pain and diarrhea was admitted to our hospital on April 23, 2008. At the age of 12, she was diagnosed with SLE based on symptoms of malar rash, discoid rash, photosensitivity, leukopenia, and a high titer of anti-nuclear antibody. She had episodes of rash, abdominal pain and hypocomplementemia. She was treated with corticosteroid and other immunosuppressants (mizoribine or cyclosporine), but these proved inadequate in controlling her SLE activities. At the age of 16, she developed lupus nephritis (World Health Organization class II). One year prior to the current admission, she presented with fever, abdominal pain and diarrhea, and a diagnosis of lupus enteritis was made. She was treated with high-dose corticosteroid including pulse methylprednisolone and with monthly intravenous cyclophosphamide (500 mg per month). Although she recovered rapidly, her serum level of complement decreased as the prednisolone was tapered to 23 mg per day.

**Figure 1:**
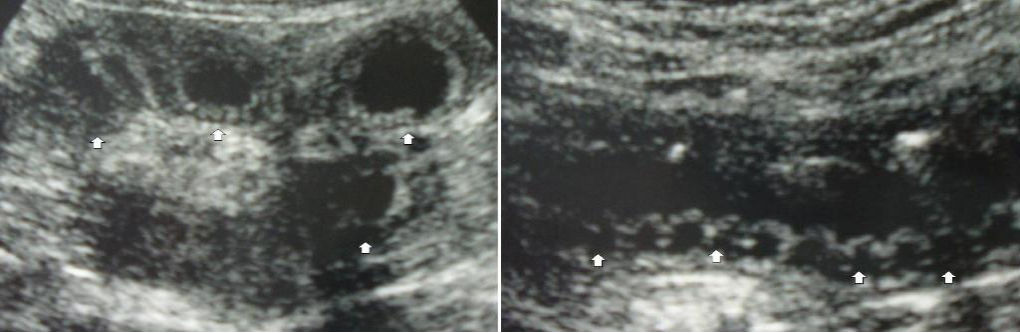
**Ultrasonography of the abdomen**. **(A) **Left image shows dilated bowel, diffuse bowel wall thickening (arrow). **(B) **Right image shows submucosal edema (arrow).

**Figure 2:**
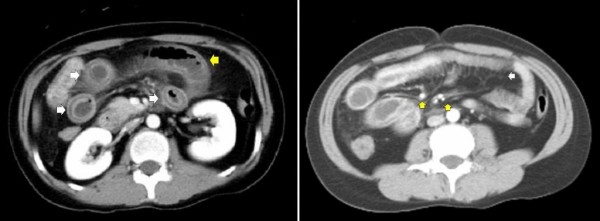
**Computed tomography scans of the abdomen**. **(A) **Left image shows distension of the bowel (yellow arrow), diffuse bowel wall thickening (maximum of 8.4 mm), abnormal bowel wall enhancement (double halo or target sign, white arrow). **(B) **Right image shows mesenteric edema, engorged mesenteric vessels (yellow arrow), prominence of mesenteric vessels with a palisade or comb-like arrangement (comb sign, white arrow). Pneumatosis cystoides intestinalis was absent.

From April 20, 2008, our patient had gastrointestinal symptoms, including watery diarrhea and abdominal pain. On admission, she was not pregnant, had a clear consciousness, and had a body temperature of 36.7°C, pulse rate of 84 beats/min, and blood pressure of 108/74 mmHg. A malar rash was also found conspicuously. Her chest and cardiovascular examination revealed no abnormalities. She showed a generalized rigidity of the abdomen with rebound tenderness and no bowel sound. Her lower extremities had no pitting edema. Meanwhile, her laboratory examination revealed leukocytosis, elevated lactate dehydrogenase and D-dimer levels, and low complement activity (Table 1). Results of her anti-dsDNA antibody, anti-cardiolipin antibody, lupus anticoagulant, anti-beta2-glycoprotein I antibody, proteinase-3-antineutrophil cytoplasmic antibody, myeloperoxidase-antineutrophil cytoplasmic antibody tests were all negative. Her electrocardiographic examination showed a regular sinus rhythm. Her chest X-ray showed neither abnormal shadow nor pleural effusion. An X-ray film of her abdomen revealed distention of her small bowel and multiple fluid levels, but no free air beneath her diaphragm was found. Her ultrasonography revealed the presence of ascites, dilated bowel, diffuse bowel wall thickening, and submucosal edema (Figure 1). Computed tomography (CT) scans of her abdomen revealed ascites, distension of the bowel, diffuse bowel wall thickening (maximum of 8.4 mm), abnormal bowel wall enhancement, and mesenteric vessels with comb-like appearance, but pancreatic enlargement, hydronephrosis, and cystitis were not observed (Figure 2).

**Table 1:** Laboratory findings on admission

Urine analysis		AMY (U/l)	67
Protein	3+	Lipase (U/l)	30
Occult blood	1+	TP (g/dl)	6.2
WBC (/μl)	15100	Alb (g/dl)	3.6
Seg. (%)	85	BUN (mg/dl)	14
Stab. (%)	0	Cr (mg/dl)	0.5
Eosi. (%)	0	Na (mEq/l)	141
Baso. (%)	0	K (mEq/l)	4.0
Ly. (%)	5	Cl (mEq/l)	103
Mono. (%)	0	SAA (μg/ml)	26.1
RBC (10^4^/μl)	589	CRP (mg/dl)	0.4
Hb (g/dl)	16.2	IgG (mg/dl)	782
PLT (10^4^/μl)	28.8	IgA (mg/dl)	93
APTT (s)	20.6	IgM (mg/dl)	37
Fib (mg/dl)	329	C3 (mg/dl)	59
D-dimer (< 0.5 mg/ml)	4.1	C4 (mg/dl)	6.5
T-Bil (mg/dl)	0.9	CH50 (U/ml)	20.1
AST (IU/l)	43	Anti-dsDNA (<12 IU/ml)	9.7
ALT (IU/l)	54	Anti-cardiolipin (< 10 U/ml)	4.8
LDH (IU/l)	495	Lupus anticoagulant(<1.3 sec)	1.1
ALP (IU/l)	213	Anti-beta2-GPI(<3.5 U/ml)	<1.3
γ-GTP (IU/l)	58	PR3-ANCA (< 3.5 U/ml)	0.4
ChE (IU/l)	518	MPO-ANCA (< 9 U/ml)	0.9

Our patient's systemic lupus erythematosus disease activity index (SLEDAI) score was 4. She received intravenous hyperalimentation and was treated with prednisolone (1.5 mg/kg/day) and cefmetazole sodium (2 g/day). Her nausea, diarrhea and abdominal pain subsided within a few days. Nine days after admission, her intestinal gas disappeared. However, her serum amylase and lipase levels were elevated in the absence of abdominal pain and her serum complement level decreased further (lowest C3, 32 mg/dl; C4, 3.1 mg/dl; CH50, <10 U/ml). As cyclophosphamide could not prevent the recurrence of her disease, we then adopted tacrolimus as an immunosuppressant. After administering tacrolimus, her serum amylase, lipase and complement levels gradually improved to within the normal range. No recurrence has yet been observed as of February 2009 despite a tapering of her prednisolone dose. Our patient is currently receiving daily doses prednisolone and tacrolimus at 18 mg and 3 mg, respectively. Her complement levels are as follows: C3, 61 mg/dl; C4, 7.3 mg/dl; CH50, 25.7 U/ml.

## Discussion

Abdominal pain is a common problem in patients with SLE, with an incidence rate of 30% to 87% [5]. These abdominal symptoms result from a variety of disorders, such as central nervous system involvement, uremia, primary peritonitis, bacterial peritonitis, acute pancreatitis, ulcer, ileus, protein-losing enteropathy, Crohn's disease, ulcerative colitis, tuberculous colitis, cytomegalovirus infection, eosinophilic gastroenteritis, and a variable degree of bowel ischemia [2,4]. The differential diagnosis for patients with SLE who present with abdominal pain should be performed quickly because all of the above disorders lead to poor prognosis. If free air, moderate amount of free fluid, acidosis, or hyperamylasemia without pancreatitis is present, early laparotomy should be considered [6].

Hizawa *et al. *assessed patients with SLE involving the small bowel by using double-contrast radiography of the duodenum and small intestine. They divided lupus-associated enteropathy into two types: an acute ischemic enteritis type and a protein-losing enteropathy type [7]. "Lupus enteritis" usually means the former, which is characterized by acute onset and severe submucosal edema, while the latter results in hypoproteinemia.

Lupus enteritis is one of the most serious complications of SLE because it may result in significant morbidity and mortality. The prevalence of lupus enteritis in patients with SLE ranges from 0.2% to 2%. Among patients with SLE who also present with acute abdominal symptoms, its occurrence has been reported to range from 45% to 79% [2,8]. The main pathophysiological features of lupus enteritis may be a result of small vessel arteritis and venulitis [8]. Associated findings include atrophy and degeneration of the media of small arteries, fibrinoid necrosis of the vessel walls, old thrombosis, phlebitis, and monocyte infiltration in the lamina propria [9].

Common CT findings in mesenteric ischemia include dilated bowel, focal or diffuse bowel wall thickening, abnormal bowel wall enhancement (double halo or target sign), mesenteric edema, engorged mesenteric vessels, and ascites [10]. The segments of bowel wall thickening were multifocal and not confined to a single vascular territory. Pneumatosis intestinalis, although very rare, has been documented [2]. The conspicuous prominence of mesenteric vessels with a palisade or comb-like arrangement (comb sign) may be an early sign of lupus enteritis. Ultrasonography (US) can demonstrate edematous thickening of the small intestine, where the submucosal layer is observed as a prominent hypoechoic area and Kerckring folds with submucosal edema and an accordion-like appearance [11]. These abnormal CT and/or US findings rapidly improve when immunosuppressive therapy becomes successful. The clinical features of the case we present were consistent with those of lupus enteritis.

Lupus enteritis often recurs. Kim *et al. *reported that there were no differences in demographic or laboratory indices, including autoantibody profiles and SLEDAI scores, between patients with non-recurrent and recurrent lupus enteritis. However, in patients with non-recurrent lupus enteritis, the cumulative dose of prednisolone and the duration of treatment with prednisolone were significantly higher than in patients with recurrent lupus enteritis. The wall thickness in patients with recurrent lupus enteritis was greater, and those with a value of >9 mm showed recurrence [3]. Kishimoto *et al. *reported two patients with recurrent lupus enterocolitis accompanied by significant hypocomplementemia and suggested acute gastrointestinal distress syndrome as a result of leuko-aggregation and gut capillary leak syndrome [12]. We also observed hypocomplementemia in our patient. However, no significant correlations between serum complement level in lupus enteritis and the occurrence of acute abdominal symptoms have been reported [8].

The treatment of severe systemic vasculitis is well established with pulsed methylprednisolone at a dose of 1 to 2 mg/kg/day in addition to complete bowel rest and administration of intravenous fluids [9]. Although there have been reports of intestinal infarction and perforation requiring emergency surgery, there have also been a number of case reports describing the successful treatment of intestinal vasculitis with high-dose prednisolone only [9]. In 1988, Laing reported the first successful treatment of corticosteroid-resistant gastrointestinal vasculitis due to SLE with pulse cyclophosphamide [13]. Grimbacher *et al. *also reported sustained remission following intravenous cyclophosphamide pulse in a patient with a severely relapsing intestinal vasculitis, which was not prevented with the administration of high-dose prednisolone [4]. Although statistical significance was not proven, patients who received intravenous cyclophosphamide showed a trend towards better outcome in the recurrences of their gastrointestinal syndrome. Our patient initially responded well to high-dose prednisolone but she demonstrated steroid dependence. Intravenous cyclophosphamide was not able to prevent the recurrence of her lupus enteritis.

In some patients whose SLE is not controlled well by conventional treatments, tacrolimus has been reported to be a useful alternative immunosuppressive agent. Maruoka *et al. *described a patient with lupus cystitis presenting with vomiting and diarrhea that was resistant to cyclophosphamide and other immunosuppressants. Tacrolimus induced remission without significant adverse events [14]. Although our patient had no symptom of cystitis, lupus enteritis and lupus cystitis often coexist, suggesting that the pathological process may be closely associated [15]. We could taper steroid gradually without inducing a relapse by using tacrolimus.

## Conclusion

Lupus enteritis is one of the most serious complications of SLE because it may result in significant morbidity and mortality. Tacrolimus can be a useful agent for the treatment of recurrent lupus enteritis that is resistant to conventional therapy, including intravenous cyclophosphamide pulse.

## Abbreviations

Alb: albumin; ALT: alanine aminotransferase; ALP: alkaline phosphatase; AMY: amylase; ANCA: anti-neutrophil cytoplasmic antibodies; Anti-beta2-GPI: anti-beta2-glycoprotein I; APTT: activated partial thromboplastin time; AST: aspartate aminotransferase; BUN: blood urea nitrogen; CH50: 50% hemolytic complement activity; ChE: cholinesterase; Cr: creatinine; CRP: C-reactive protein; Fib: fibrinogen; FDP: fibrin degradation product; Ig: immunoglobulin; LDH: lactate dehydrogenase; MPO: myeloperoxidase; PR3: protenase3; PLT: platelets; RBC: red blood cells; SAA: serum amyloid protein A; T-Bil: total bilirubin; TP: total protein; WBC: white blood cells; γ-GTP: γ-glutamyl-transpeptidase.

## Consent

Written informed consent was obtained from the patient for publication of this case report and any accompanying images. A copy of the written consent is available for review by the Editor-in-Chief of this journal.

## Competing interests

The authors declare that they have no competing interests.

## Authors' contributions

TS was a major contributor in writing the manuscript. YH revised and help to write the manuscript. TS, YH, RW, YT, HF, NT, TI, and HH treated the patient. All authors read and approved the final manuscript.
